# Gloomy and out of control? Consequences of the COVID-19 pandemic on momentary optimism in daily lives of adolescents

**DOI:** 10.1007/s12144-022-03313-6

**Published:** 2022-06-25

**Authors:** Larissa L. Wieczorek, Eva Bleckmann, Naemi D. Brandt, Jenny Wagner

**Affiliations:** Department of Educational Psychology and Personality Development, Institute of Psychology, University of Hamburg, Hamburg, Germany

**Keywords:** Momentary optimism, Perceived control, Adolescence, COVID-19

## Abstract

In the global COVID-19 pandemic, adolescents are regarded as especially burdened due to school closures and leisure activities being banned, often reducing peer contacts to zero. Experiencing restrictions while being uninvolved in decision-making processes left them with little control over their daily lives. Meanwhile, research highlights that optimism can act as a buffer against the impact of daily hassles and is considered an important resource for mental health. To understand the implications of the COVID-19 pandemic for adolescents' lives, this study examined how momentary perceived control and perceived personal and societal consequences of the COVID-19 pandemic relate to momentary optimism. Using experience-sampling data from *N* = 242 (*M*_age_ = 15.89; 86% female) adolescents assessed during the second pandemic wave in Germany, multilevel modeling revealed positive associations between adolescents’ momentary perceived control and their momentary optimism at both the within- and between-person level. Additionally, perceived consequences of the COVID-19 pandemic showed specific associations with momentary optimism: Whereas perceived societal consequences were directly related to lower momentary optimism, perceived personal consequences strengthened the relationship between momentary perceived control and optimism. These findings highlight the role of perceived control as an important resource for optimism both as a general tendency and within specific situations in daily life. This way, our results may shed light on how to support adolescent optimism during states of emergency, such as a worldwide pandemic.

**Supplementary Information:**

The online version contains supplementary material available at 10.1007/s12144-022-03313-6.

## Introduction

The COVID-19 pandemic has changed people's daily lives drastically from one day to another. Worldwide, governments imposed restrictions on movement and social contacts (Brooks et al., 2020; Ritchie et al., 2020), and people were confronted with the fear of infection, disease, and death (Mertens et al., 2020). Although being oftentimes neglected in the political and media debates, state-mandated containment measures particularly changed the life of adolescents. Apart from schools closing their doors for months, leisure activities and peer contacts were often reduced to zero (Branje & Morris, 2021; Kovacs et al., 2021; Rajabi et al., 2021), diminishing individuals’ control over their daily lives. Empirical evidence starts to accumulate that younger cohorts were especially burdened by the containment measures: For instance, adolescents reported increases both in anxiety and depressive symptoms, as well as decreases in their general life satisfaction (Magson et al., 2021; Rogers et al., 2021). In line with these findings, initial research based on an adult sample (Arslan et al., 2020a) also suggests a decline in optimism relating to the experiences during the COVID-19 pandemic. Optimism—defined as the general tendency to expect positive outcomes (Carver et al., 2010; Peterson, 2000)—is a key ingredient for a happy life. In previous research, optimism has been linked to both positive mental and physical health (e.g., Peterson & Bossio, 2001; Rincón Uribe et al., 2021), more successful coping with stress (Reed, 2016), and more satisfying social relationships (Monzani et al., 2014; Srivastava et al., 2006). Whereas optimism has proven as a major resource during the pandemic (Genç & Arslan, 2021; Reizer et al., 2022), little is known about the perceptions and experiences that contribute to it in daily life. To address this research gap, this study aimed to examine how adolescents’ momentary optimism relates to situational perceptions of control in their daily lives and to more general perceptions of the consequences of the COVID-19 pandemic.

### Optimism and perceived control in daily life

Although optimism is most commonly assessed as a relatively stable trait, previous research has revealed that it is also subject to change (Chopik et al., 2020; Schwaba et al., 2019) and even fluctuates over short periods of time (e.g., on a daily basis; Shifren & Hooker, 1995). Defined as the specific expectation of positive outcomes within a situation (Peterson, 2000), *momentary optimism* has been linked with more positive outcomes in daily life, such as better coping with acute interpersonal conflict or job-related stress (Kluemper et al., 2009; Martinez-Corts et al., 2015). Given that momentary optimism represents an important resource protecting adolescents against daily hassles (Rincón Uribe et al., 2021), it is important to gain a better understanding of the factors that contribute to momentary optimism, particularly in the context of the worldwide COVID-19 pandemic.

Ample research on the trait level has shown that optimism is positively associated with an individual’s perceived control (Gillham & Reivich, 2004; Röcke & Lachman, 2008). Perceived control refers to the belief that one can determine one’s own internal states and behaviors, and that one’s own behaviors will have an impact (Bandura, 1997; Wallston et al., 1987). Both theoretical (Abramson et al., 1978; Gillham & Reivich, 2004; Peterson, 2000) and empirical research (Röcke & Lachman, 2008; Scholz & Strelan, 2021; Sherman & Cotter, 2013) have suggested that perceiving higher control nourishes a higher sense of optimism. Corroborating this assumption, a recent longitudinal study demonstrated that a higher sense of control at age 14 predicted optimism at age 18 (Renaud et al., 2019). The link between perceived control and optimism may be especially important in adolescence. During this developmental phase, individuals become increasingly independent and develop a sense of personal control over their lives (Petito & Cummins, 2000; Schulz & Heckhausen, 1996). Moreover, previous research has shown that perceived control is—as optimism—malleable and can fluctuate throughout the day and across situations (Koffer et al., 2019). Given the established association between trait control and optimism, daily perceptions of low control might also contribute to lower levels of adolescents’ momentary optimism.

### Adolescents’ perceived consequences of the COVID-19 pandemic

The pandemic has affected adolescents’ lives in various domains. As a part of state-mandated containment measures, schools have been closed or operated with intermittent lessons and social contacts were reduced to a minimum of other households and people (Bundesrechtsanwaltskammer, 2021). In addition, policy- and media-driven debates have focused on the pandemic for months, raising public awareness of its impact on health, the economy, and society (Gozzi et al., 2020; Pearman et al., 2021). Together, adolescents have faced consequences of the pandemic both in their personal life (e.g., perceiving a disrupted school routine) and at a larger, societal level (e.g., perceiving how the society copes with COVID-19). Accordingly, we assessed adolescents’ perceived personal and societal consequences of the COVID-19 pandemic (see Back et al., 2020).

Initial findings suggest that individual experiences during the ongoing COVID-19 pandemic are negatively related to people's optimism. Specifically, Arslan et al. (2020a) found that higher levels of COVID-19-related stress were negatively associated with trait optimism in an adult sample. Accordingly, holding more negative views on personal and societal consequences of the COVID-19 pandemic could manifest in lower levels of momentary optimism in adolescents’ daily lives.

In addition to the association with momentary optimism, adolescents’ perceptions of the pandemic might also impact the link between perceived control and optimism. Specifically, the restrictions imposed by the government have limited the individual’s scope of action and therefore might have also lowered the degree of perceived control in their daily lives (see Goodwin et al., 2021). For example, meeting a group of friends was prohibited during lockdown phases and strongly limited during the entire pandemic as part of social distancing measures (Bundesrechtsanwaltskammer, 2021). Given the limited possibilities to exert control in peoples’ daily lives during the COVID-19 pandemic, the association between momentary perceived control and optimism might have been weakened, especially for adolescents who perceived more severe personal and societal consequences. To date, however, no study has explored if and how the link between perceived control and momentary optimism is moderated by an individual's perceptions of the pandemic.

### The present study

Using experience sampling data of *N* = 242 adolescents living in Germany assessed during the second wave of the COVID-19 pandemic, this study had two goals. First, we aimed to understand whether adolescents’ momentary perceptions of control during the pandemic are related to their momentary optimism. Building on previous research (Scholz & Strelan, 2021; Sherman & Cotter, 2013), we expected that higher levels of momentary perceived control within daily situations predict higher levels of momentary optimism. Second, we explored whether adolescents’ perceived consequences of the COVID-19 pandemic moderate the link between perceived control and momentary optimism. Specifically, given that state-mandated containment measures have not only reduced an individual’s scope of action but most likely also have lowered the perception of control across daily situations in adolescents (Goodwin et al., 2021), we assumed that more negative perceptions of the pandemic’s consequences dampen the link between momentary perceived control and momentary optimism.

Building on rich theoretical work (Abramson et al., 1978; Gillham & Reivich, 2004; Peterson, 2000) and initial empirical findings (Scholz & Strelan, 2021; Sherman & Cotter, 2013) on the association between perceived control and optimism, the present study extends previous research in at least three important ways. First, by using the experience sampling method (ESM), our study provides insights into the momentary associations between perceived control and optimism that occur in adolescents’ everyday lives. Second, by using data collected during the second wave of the COVID-19 pandemic, the present study zooms in on a unique historical and societal phase in Germany that allows to investigate associations between perceived control and optimism under particularly challenging conditions. Finally, by examining the association between perceived control and optimism in an adolescent sample, we shed light on a life phase that is assumed to form the basis for sense of control in adulthood (Petito & Cummins, 2000; Schulz & Heckhausen, 1996). At the same time, adolescents have been especially burdened by the COVID-19 pandemic (Magson et al., 2021; Rogers et al., 2021) and our study is one further step to better understand interindividual differences in the consequences that might result for their daily optimism.

## Method

This study is the first using data from the School and Life during Corona (SchoCo) project (https://osf.io/r5gjx/), a longitudinal and intensive investigation of the development of personality traits, social relationships, and educational outcomes during the second wave of the COVID-19 pandemic in Germany. Ethical approval for the SchoCo project was granted by the ethics committee of the Faculty of Psychology and Human Movement Science at the University of Hamburg. The current study used data from the first measurement point and the first experience sampling period only (November 12 – December 23, 2020). During this time, contact restrictions were tightened and public life increasingly shut down (e.g., restaurants and shops closed), while schools remained open until early to mid-December (Bundesrechtsanwaltskammer, 2021). The study was promoted via social media platforms, personal outreach to schools and teachers, and leaflets in schools to attract potential participants from the target age group. Participants completed an online questionnaire implemented with the open-source software formr (Arslan et al., 2020b), reporting, among other variables, their perceptions of the pandemic situation and basic demographics. A day after this first questionnaire, participants were invited to a one-week experience sampling period. During this week, participants received a short questionnaire on their smartphones five times a day (9 a.m., 12 p.m., 3 p.m., 6 p.m., and 8 p.m.) including questions about adolescents’ momentary perceived control and their momentary optimism. As an incentive for participation, adolescents entered a lottery after each measurement point to win gift vouchers and sweets. In addition, all participants received personalized feedback after completing the entire study.

### Participants

Within SchoCo, a convenience sample of 304 adolescents completed the first online questionnaire. Prior to analyses, we excluded participants who did not take part in the ESM assessments (*n* = 62) and who had ESM entries with zero variance across all ratings (*n* = 1). The only inclusion criterion for the study was being between 14 and 18 years old, however, we did not exclude the one participant who was already 19 at the time of data collection. Thus, in our final sample, *N* = 242 adolescents (86% female) aged 14–19 years (*M* = 15.89, *SD* = 1.21) provided an average of 12.33 (*SD* = 9.83, Range: 1–35) ratings during the ESM week. This resulted in a total of 2,985 ESM entries. To approximate the socioeconomic status, participants were asked to indicate the education of their father and mother: About 28% (24%) reported that their father (mother) had graduated from university, 27% (29%) indicated that their father (mother) had completed high school or an apprenticeship. Regarding their own educational background, 78% of the participants indicated that they were attending high school at the time of the study. Selectivity analyses based on Welch's *t*-tests revealed that the final sample and the excluded participants did not differ statistically significantly from each other in any variable of the study (i.e., age, gender, perceived personal and societal consequences, or contact with COVID-19 infections).

Since no previous studies exist that allow to derive expected effect sizes, we conducted a simulation-based sensitivity analysis using the R-package "simr" (Green & MacLeod, 2016), following instructions by Arend and Schäfer (2019). To this end, we fitted two-level multilevel models with one predictor on each level, representing the basic structure of our main models. We used approximate sample sizes and estimated power for varying unstandardized effects at both Level 1 and Level 2 of the multilevel model. This way, we sought to assess how well our models given our sample sizes at Level 1 and Level 2 could detect effects of different sizes. The simulation of unstandardized multilevel effects indicated sufficient power to detect within-person (Level 1) effects of *b* = 0.10, between-person (Level 2) effects of *b* = 0.15, and cross-level interactions of *b* = 0.20. More details can be found in our preregistration at the OSF.

### Measures

The SchoCo study included a wide range of questionnaires and ESM items (for an overview, see https://osf.io/r5gjx/). In the following, we will only introduce those measures that were relevant to the current study.

#### Momentary optimism and perceived control

During the ESM period, participants provided momentary ratings of optimism (“How optimistic do you feel right now?”) and perceived control (“How much do you feel like you can control what is happening in your life right now?”) a maximum of five times a day. These items were rated on a scale ranging from 1 (*not at all*) to 10 (*very*). The item for perceived control was adapted from a previous ESM study (Wagner et al., 2021). In ESM studies, the intraclass correlation coefficient (ICC) type 2 can be used to assess the reliability of an individual’s mean score across their ESM ratings (Lüdtke & Trautwein, 2007). Based on an average of 12.33 ESM ratings, the ICC(2) displayed satisfactory reliabilities of 0.87 for momentary optimism and 0.92 for momentary perceived control.

#### Perceptions of the COVID-19 pandemic

To assess adolescents’ perceived consequences of the COVID-19 pandemic, we used an adapted version of items from the Coping with Corona (CoCo) study (Back et al., 2020). With these items, individuals were asked to report their perceptions of personal and societal consequences relating to the current situation once. Perceptions of personal consequences of the COVID-19 pandemic were measured with an aggregate of four items following the format “I currently [personal consequence]”. Personal consequences included having fewer contacts with friends, having trouble focusing on schoolwork, avoiding social contacts out of fear of infection, and doing well with avoiding contact with others (reversed). Participants rated their level of agreement with the item on a scale ranging from 1 (*strongly disagree*) to 7 (*strongly agree*). Internal consistency across the four items measuring personal consequences as indicated by total omega was 0.54. Perceptions of societal consequences of the COVID-19 pandemic were measured with an aggregate of two items following the format “Concerning the Coronavirus and its containment [societal consequence]”. Societal consequences included being anxious/worried, and believing that we as a society can get the situation under control (reversed), and were rated on a scale ranging from 1 (*strongly disagree*) to 7 (*strongly agree*). Given that total omega cannot be computed as an estimate of internal consistency for two-item measures, we calculated the Spearman-Brown correlation between the two items measuring societal consequences, which was 0.26.

#### Control variables

At the beginning of the study, participants reported their age in years and were asked whether they identified themselves as female (coded as 0) or male (coded as 1). In addition, they indicated whether they or any person close to them have had personal contact with COVID-19 infections. Specifically, participants indicated whether one of the following persons had been infected by the coronavirus: (a) the participants themselves, (b) a family member, (c) a close friend. If none of these items were answered with “yes”, personal contact with COVID-19 infections was coded as no contact (0). Conversely, if one of these items was answered with “yes”, personal contact with COVID-19 infections was coded as contact (1).

### Data analysis

Data cleaning, structuring, and all analyses were performed with R Version 4.0.2 (R Core Team, 2020), using RStudio (RStudio Team, 2021). Our hypotheses and all data analytical steps were preregistered on OSF before data cleaning and analyses began (https://osf.io/kbdfz). To account for the nested data structure (measurement points nested in individuals), we estimated linear multilevel regression models specified on two levels using the *lme4* package (Bates et al., 2015): within-person (Level 1) and between-person (Level 2). Perceived control as a within-person predictor was centered at the participants’ individual mean, whereas all continuous between-person predictors were centered at the sample mean (Bolger & Laurenceau, 2013) using the *misty* package (Yanagida, 2021). To answer our outlined research questions, we first fitted a model with momentary perceived control at the within-person level. Additionally, we added the individual mean of perceived control across all ESM entries as an additional predictor at Level 2 to consider that the effects of momentary perceived control might also manifest between persons. Subsequently, we extended this model by fitting two separate models for the two types of perceived consequences of the COVID-19 pandemic (i.e., personal vs. societal consequences). More details on the basic model setup can be found in our preregistration on OSF.

Extending these models, we included several control variables. Following the recommendation to consider the time of assessment in ESM research (Bolger & Laurenceau, 2013), we included the day of the ESM (ranging between 0 for day 1 to 6 for day 7) and weekday (coded as 0) vs. weekend (coded as 1) as control variables at the within-person level. At the between-person level, we included the participants’ age, gender, and personal contact with COVID-19 infections.

For all models, we report exact *p*-values together with 95% confidence intervals and discuss all effects that are statistically significant at *p* < 0.05. We furthermore calculated ${R}_{w}^{2}$ and ${R}_{b}^{2}$, reflecting the proportional reduction in the mean squared prediction error at the within-person level and the between-person level, respectively, as estimates for the amount of explained variance in each model (Snijders & Bosker, 1994). The code and the data that are necessary to reproduce all results and supplementary materials can be retrieved from https://osf.io/bgrzw/.

## Results

Descriptive statistics and intercorrelations of the within- and between-person variables are presented in Tables 1 and 2. As indicated by the ICC(1), 50% of the variance of momentary perceived control and 36% of the variance in momentary optimism was on the between-person level. Thus, most of the variance of momentary optimism was at the within-person level, illustrating a substantial amount of fluctuation in optimism across daily life. Variance in momentary perceived control was evenly distributed across different situations within one person and differences between persons. At the within-person level, momentary control beliefs correlated positively with momentary optimism (*r* = 0.50), suggesting that in situations in which individuals felt in control, they also reported higher levels of optimism (see Table 1). As illustrated in Table 2, aggregated momentary optimism negatively correlated with personal (*r* = –0.17) and societal (*r* = –0.21) consequences of the pandemic at the between-person level, indicating that optimism aggregated across situations was lower when individuals perceived the pandemic’s consequences as more severe in either domain. Finally, personal and societal consequences were positively correlated (*r* = 0.26), suggesting that individuals who perceived their personal consequences of the pandemic as more severe also did so regarding societal consequences.

**Table 1:** Intercorrelations among within-person variables

Variable	*M*	*SD*	1	2	3
1. Optimism	5.27	2.59			
2. Perceived control	5.56	2.87	0.50*		
3. Weekend	0.29	0.45	0.01	0.03	
4. Day of ESM	2.46	1.97	0.03	0.03	0.09*

*N* = 2,985 observations nested in 242 individuals. * *p* < 0.05

**Table 2:** Intercorrelations among between-person variables

Variable	*M*	*SD*	1	2	3	4	5	6
1. Optimism	5.15	1.90						
2. Perceived control	5.39	2.23	0.58*					
3. Personal consequences	4.17	1.04	-0.17*	-0.19*				
4. Societal consequences	3.85	1.29	-0.21*	-0.20*	0.26*			
5. Age	15.89	1.21	0.03	-0.10	0.10	0.11		
6. Gender (1 = male)	0.14	0.35	0.15*	0.08	-0.01	-0.01	-0.03	
7. Contact with COVID-19 (1 = yes)	0.22	0.41	0.05	0.09	0.08	-0.04	0.01	-0.02

*N* = 242 individuals. Situational variables (momentary optimism, momentary perceived control) were averaged across measurements for each individual and then aggregated to a sample mean across individuals. * *p* < 0.05

### Perceived control and momentary optimism

The results of the two multilevel models are reported in Table 3. Our first research hypothesis focused on the link between momentary perceived control within daily situations and an individual’s level of momentary optimism. As expected, momentary perceived control was consistently related to momentary optimism across both models ($\gamma$_10_ = 0.44, *p* < 0.001). That is, adolescents reported higher momentary optimism in daily situations where they also perceived higher personal control. In addition to this within-person association, perceived control at the between-person level was also consistently associated with momentary optimism (range $\gamma$_01_ = 0.46 – 0.47, *p*s < 0.001): Adolescents who reported higher levels of perceived control across situations also experienced higher momentary optimism within specific situations.

**Table 3:** Multilevel models with momentary optimism as outcome

Variables	Personal Consequences			Societal Consequences		
	*Est*	*95% CI*	*p*	*Est*	*95% CI*	*p*
Fixed Effects						
Intercept, ${\gamma }_{00}$	5.15	[4.92, 5.37]	< 0.001	5.16	[4.94, 5.38]	< 0.001
*Within-person effects*						
Perceived control_WP_, ${\gamma }_{10}$	0.44	[0.38, 0.49]	< 0.001	0.44	[0.38, 0.49]	< 0.001
Weekend, ${\gamma }_{20}$	-0.04	[-0.19, 0.12]	0.649	-0.04	[-0.19, 0.12]	0.648
Day of ESM, ${\gamma }_{30}$	0.02	[-0.02, 0.06]	0.264	0.02	[-0.02, 0.06]	0.267
*Between-person effects*						
Perceived control_BP_, ${\gamma }_{01}$	0.47	[0.39, 0.56]	< 0.001	0.46	[0.38, 0.55]	< 0.001
Pandemic consequences, ${\gamma }_{02}$	-0.10	[-0.28, 0.08]	0.281	-0.15	[-0.29, -0.01]	0.032
Age, ${\gamma }_{03}$	0.12	[-0.03, 0.27]	0.121	0.13	[-0.02, 0.28]	0.087
Gender, ${\gamma }_{04}$	0.51	[-0.02, 1.03]	0.058	0.51	[-0.01, 1.03]	0.053
Contact with COVID-19, ${\gamma }_{05}$	0.11	[-0.33, 0.54]	0.631	0.08	[-0.35, 0.51]	0.713
*Cross-level interactions*						
Perceived control $\times$ Pandemic consequences, ${\gamma }_{11}$	0.06	[0.01, 0.12]	0.017	0.01	[-0.03, 0.06]	0.495
Random Effects						
Variance Intercept, ${\sigma }_{{u}_{0}}^{2}$	1.47			1.44		
Variance Perceived Control ${\sigma }_{{u}_{1}}^{2}$	0.06			0.06		
Residual Variance, ${\sigma }_{e}^{2}$	3.33			3.34		
ICC	0.33			0.33		
AIC	12,591.228			12,592.986		
${R}_{\mathrm{WP}}^{2}/{R}_{\mathrm{BP}}^{2}$	0.296 / 0.378			0.300/0.389		

*N* = 242 individuals providing 2,985 observations. WP = between-person level; BP = between-person level. Gender was coded 0 for females and 1 for males, therefore the intercept refers to the female gender. ICC represents the intraclass correlation, calculated with a null model.

### The moderating role of perceived consequences of the pandemic

**Fig. 1:**
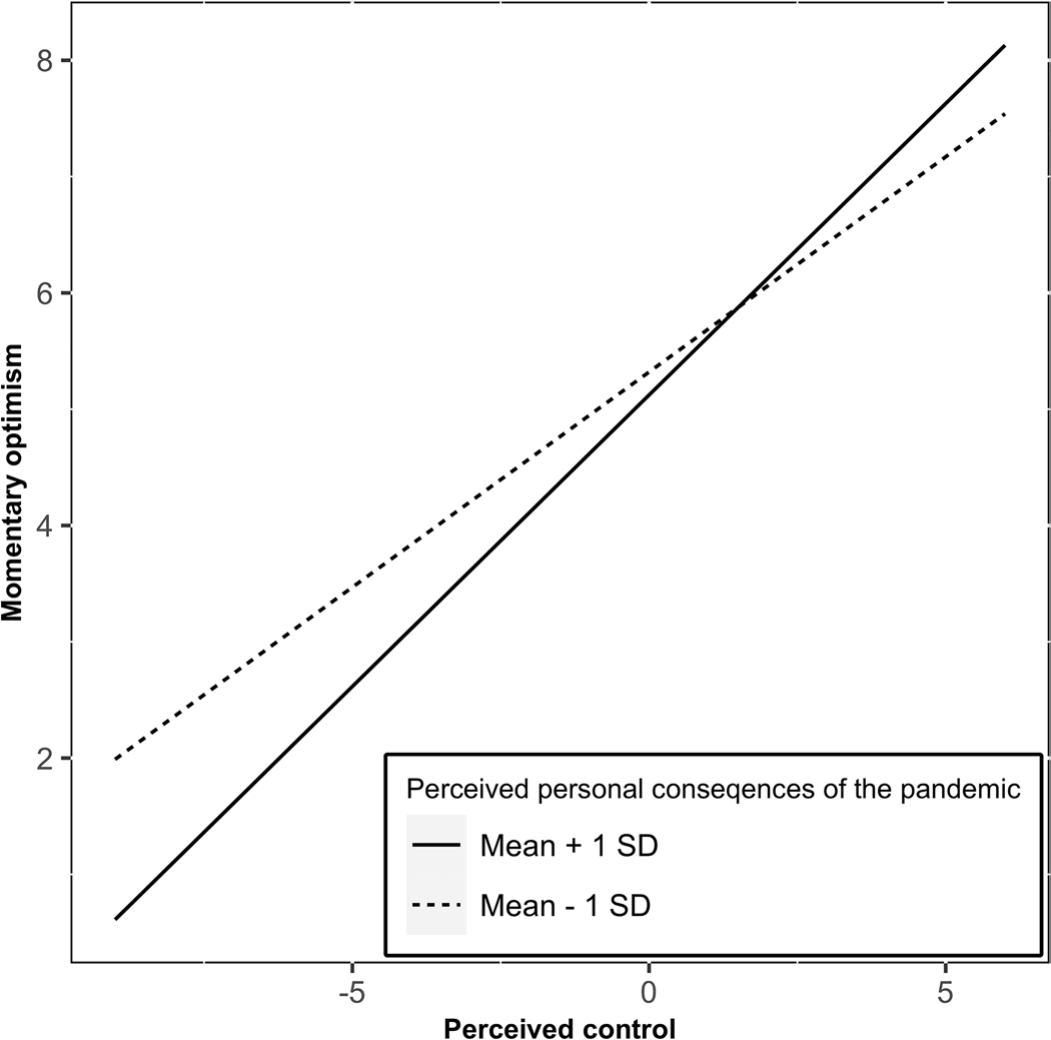
Moderating effect of perceived personal consequences of the COVID-19 pandemic on the association between perceived control and momentary optimism

Our second research question addressed the role of adolescents’ perceived consequences of the COVID-19 pandemic. Comparing perceptions of personal and societal consequences, a differential pattern appeared: First, whereas personal consequences of the COVID-19 pandemic displayed no significant associations with an individual’s momentary optimism, they moderated the link between perceived control and momentary optimism ($\gamma$_11_ = 0.06, *p* = 0.017). As displayed in Fig. 1, individuals with more negative perceptions of the pandemic’s personal consequences did not feel less optimistic but experienced especially high levels of momentary optimism in situations with higher perceived control. Second, higher perceived societal consequences were associated with lower momentary optimism ($\gamma$_02_ = – 0.15, *p* = 0.032), but did not moderate the link between momentary perceived control and momentary optimism. Thus, adolescents who perceived the societal consequences of the pandemic as more severe felt less optimistic in everyday situations, regardless of the level of control they perceived within these situations. Across both models, none of the control variables (i.e., weekday vs. weekend, day of the ESM, age, gender, or actual contact with COVID-19 infections) showed statistically significant associations with momentary optimism.

### Follow-up analyses

We conducted three follow-up analyses to explore additional explanations of our findings. First, as preregistered, we considered that actual consequences might be more salient for adolescents in their daily lives than generally perceived consequences. However, actual contact with COVID-19 was not significantly related to adolescents' momentary optimism and did not moderate the relationship between perceived control and optimism at the within-person level (see Table S1).

Second, as growing evidence suggests that extraverted individuals may have perceived the imposed social restrictions as more severe (Alt et al., 2021; Zacher & Rudolph, 2021), we explored whether extraversion would moderate the link between perceived consequences of the pandemic and momentary optimism.^1^ As shown in Table S2, extraversion was not statistically significantly related to momentary optimism and did not moderate the association between perceived consequences of the pandemic and optimism in our adolescent sample. Thus, perceptions of personal and societal consequences of the pandemic illustrated similar associations with momentary optimism among adolescents with lower and higher levels of extraversion.

Third, the two indices assessing adolescents’ perceived consequences of the pandemic had relatively low reliabilities, indicating that each of them integrated rather diverse perceptions of the pandemic. Accordingly, we included individual items in our multilevel models to investigate the potential impact of these specific perceptions in an exploratory fashion. The results of these exploratory analyses are displayed in Tables S3 and S4. Of the four items used to indicate the personal consequences of the COVID-19 pandemic, only the item "I am currently having even more trouble concentrating on my schoolwork" statistically significantly related to momentary optimism ($\gamma$_02_ = – 0.10, *p* = 0.040). Thus, adolescents who reported more problems in school generally reported lower levels of momentary optimism in daily situations. The by-item models also revealed one significant positive cross-level interaction between the reversed item “I currently do well with avoiding contact with other people” and momentary perceived control ($\gamma$_11_ = 0.04, *p* = 0.034). This suggests that adolescents who found it more difficult to avoid social contact felt especially optimistic in situations with higher perceived control. Similarly, we fitted separate models with the two items that indicated societal consequences. While the item “concerning the Coronavirus and its containment, I am anxious/worried" was not significantly linked to momentary optimism, the item “concerning the Coronavirus and its containment, I believe we as a society can get the situation under control" was negatively associated with momentary optimism ($\gamma$_02_ = – 0.24, *p* < 0.001). That is, individuals who thought that society was able to handle the pandemic also reported higher levels of optimism in daily situations. Since we did not preregister these by-item analyses, we highlight their exploratory character and consider the results to be preliminary.

## Discussion

This study examined whether momentary perceptions of control were related to momentary optimism during the COVID-19 pandemic in adolescents—a cohort particularly burdened by the current pandemic (e.g., Magson et al., 2021). Furthermore, we tested whether adolescents’ perceived consequences of the COVID-19 pandemic dampened this association. Extending previous research on trait perceived control and optimism (Scholz & Strelan, 2021; Sherman & Cotter, 2013), our results highlight that, first, momentary perceived control was strongly linked to adolescents’ momentary optimism, both within specific situations and as a general tendency to perceive control across situations. Second, our findings emphasize the adaptive capacities of human functioning as more severe consequences of the COVID-19 pandemic did not necessarily diminish adolescents’ momentary optimism. Specifically, more severe personal consequences had no direct effect on momentary optimism but strengthened the link between momentary perceived control and optimism. Thus, perceived control seemed to be especially important for momentary optimism if individuals perceived the pandemic as personally challenging. In contrast, we found lower momentary optimism among adolescents who reported more severe societal consequences irrespective of the degree of perceived control. We discuss how these findings inform opportunities to support adolescents’ optimism in daily life.

### Perceived control and optimism are associated in daily life

Whereas previous studies (Scholz & Strelan, 2021; Sherman & Cotter, 2013) focused on adult participants and stable, trait-like aspects of perceived control and optimism, our findings demonstrate that this association generalizes to an adolescent sample and within daily life. Importantly, our findings stress the need to support adolescents’ perceived control across daily situations—beyond their general perceptions of control—in order to promote momentary optimism. In everyday life, parents and teachers might foster adolescents’ perceived control by involving them in decision making, assigning responsibilities, or by providing them with structure and predictability (Infurna & Infurna, 2017). During the pandemic, clear governmental guidance and communication might additionally help adolescents to increase their perceived control and help to stay optimistic.

### Adolescents’ perceptions of the COVID-19 pandemic: Differential roles of personal and societal consequences

Consequences of the COVID-19 pandemic for adolescents’ momentary optimism resulted in a differential pattern: Whereas personal consequences of the pandemic reinforced the link between momentary perceived control and momentary optimism, societal consequences were associated with lower levels of momentary optimism but did not interact with momentary perceived control. One potential explanation might be the scope of action individuals (believe they) have in coping with the pandemic’s consequences. Adolescents reasonably expect more opportunities to change their current personal situation (e.g., use new social contact channels) but may see few possibilities to change society’s handling of the pandemic situation. Accordingly, societal consequences may pose a more direct threat to adolescents’ optimism, regardless of how much control they have over their current situation.

The explorative by-item analyses support this argumentation. Whether personal consequences moderated the link between momentary perceived control and optimism pertained to specific consequences, namely, how well they coped with the social restrictions. As such, some adolescents potentially coped with the state-mandated social restrictions by keeping social contact with their peers via social media (Ellis et al., 2020) or by compensating for reduced contact with friends through family relationships (Campione-Barr et al., 2021). In contrast, reporting trouble focusing on school tasks did not moderate the link between momentary perceived control and optimism. It directly resulted in lower levels of reported momentary optimism. Adolescents might have felt little scope of action and therefore experienced difficulties dealing with the changed school routine.

Furthermore, the by-item analyses suggested that the main effect of perceived societal consequences on momentary optimism was likely driven by adolescents’ perception of whether society is successful in controlling the pandemic situation. Accordingly, attributing control to society may be beneficial for an individual's optimism beyond the positive effect of a person’s own perceived control. This is in line with previous research showing that perceived control can be conceptualized at different levels, such as at the individual level and at the level of larger social groups (A. J. Schulz et al., 1995). To further enhance the understanding of how perceived control and optimism are related during the COVID-19 pandemic, it may be useful to consider perceived control on multiple levels.

### Limitations and future directions

Despite the strength of our study assessing adolescents’ perceptions in daily life, we also note limitations. First, the correlational nature of our data does not allow for causal conclusions. Although previous research largely assumes that perceived control is a prerequisite of optimism (Peterson, 2000; Sherman & Cotter, 2013), both constructs were assessed during the same ESM assessment. Thus, we cannot exclude the opposite direction of effects or a bidirectional association, nor can we infer any long-term associations between the two constructs. In addition, future research is needed to understand whether and how increases in momentary perceived control and optimism (i.e., at the within-person level) might translate into changes at the trait level (i.e., at the between-person level). Second, we note that other studies used more extensive measures of momentary optimism (e.g., Martinez-Corts et al., 2015). Although our single-item measures of momentary optimism and perceived control illustrated good reliability, future studies should examine their relationship with more comprehensive measures. This being said, we would like to caution that especially in ESM studies, it is important to critically consider the number of items to minimize participant burden. Third, our power was limited to detect small cross-level interaction effects. Thus, our moderation results should be seen as tentative evidence that requires replication in larger samples. Finally, large parts of our sample (86%) were female and data were restricted to the second corona wave. Consequently, the generalizability to more diverse adolescent samples and later phases of the pandemic remains unclear. Compared to the rather immediate impact of the pandemic on school-related aspects, adolescents' perceptions of pandemic consequences in their social lives might have manifested in their momentary optimism with some temporal delay. As our study assessed adolescents’ momentary optimism at a time when restrictions started to become stricter again, individuals might have benefited from the social support they have received in previous weeks (Bernasco et al., 2021). Therefore, studies investigating diverse samples and longer timespans of the pandemic should add to this picture.

## Conclusion

Did the COVID-19 pandemic diminish adolescents’ momentary optimism? This was not necessarily the case. The findings of this study suggest that perceived control—both as a general tendency and within specific, daily situations—is a key resource for optimism, potentially even more so when facing threatening circumstances such as a global pandemic. Whereas perceiving more severe societal consequences of the pandemic was related to lower momentary optimism among adolescents, perceiving personal consequences strengthened the positive association between momentary perceived control and optimism. As such, it appears that as long as adolescents have sufficient scope to act, they are able to keep a positive outlook. Altogether, our findings provide important insights into the interplay of perceived control and optimism on a momentary level. In addition, considering the beneficial effects of optimism on mental health, we hope that our study offers a promising starting point for practitioners in developing strategies that support adolescents' optimism and help them cope with the impact of the pandemic on their daily lives.

## Supplementary Information

Below is the link to the electronic supplementary material.

Supplementary file1 (PDF 499 KB)

## Footnotes

1 Extraversion was measured with the BFI-2 (Danner et al., 2019) in an online survey preceding the experience sampling period.

## Declarations

### Open practice statement

This study was preregistered at https://osf.io/kbdfz/. Open code and data are posted at https://osf.io/bgrzw/. A codebook of the SchoCo study including all study materials can be found at https://osf.io/r5gjx/.
