# Supplementary material for: Gloomy and out of control? Consequences of the COVID-19 pandemic on momentary optimism in daily lives of adolescents
Source: Curr Psychol. 2022 Jun 25:1–11. Online ahead of print. doi: 10.1007/s12144-022-03313-6 (PMC9244014; doi:10.1007/s12144-022-03313-6)
Supplement: Supplementary file 1 — Supplementary file1 (PDF 499 KB) [file 12144_2022_3313_MOESM1_ESM.pdf]

Supplement

Table S1

Exploratory Analyses With Contact With COVID-19 as a Moderator of the Link Between Momentary Perceived Control and Momentary Optimism

Variables	Model Parameters		
	Est.	*95% CI*	*p*
**Fixed Effects**			
Intercept, γ_{00}	5.16	[4.94, 5.38]	< .001
Within-person effects			
Perceived control$_{WP}$, γ_{10}	0.45	[0.39, 0.52]	< .001
Weekend, γ_{20}	- 0.04	[-0.19, 0.12]	.644
Day of ESM, γ_{20}	0.02	[-0.02, 0.06]	.259
Between-person effects			
Perceived control$_{BP}$, γ_{01}	0.48	[0.40, 0.57]	< .001
Age, γ_{02}	0.11	[-0.04, 0.26]	.140
Gender, γ_{03}	0.51	[-0.02, 1.03]	.060
Contact with COVID-19, γ_{03}	0.08	[-0.35, 0.51]	.720
Cross-level interactions			
Perceived Control × Contact with COVID-19, γ_{11}	- 0.07	[-0.20, 0.06]	.294
**Random Effects**			
Variance Intercept, $\sigma^2_{u_0}$			1.48
Variance Perceived Control $\sigma^2_{u_1}$			0.06
Residual Variance, σ^2_e			3.33
ICC			.34
AIC			12594.899
R^2_w/ R^2_b			.296 /.378

Note. *N* = 242 individuals providing 2,985 observations. Gender was coded 0 for females and 1 for males. R^2_windicates modeled variance at the within-person level, R^2_b indicates modeled variance at the between-person level.

Table S2

Exploratory Analyses with Extraversion as a Moderator of Perceptions of the Pandemic

Variables	Personal Consequences			Societal Consequences		
	Est.	*95% CI*	*p*	*Est.*	*95% CI*	*p*
**Fixed Effects**						
Intercept, γ_{00}	5.18	[4.95, 5.40]	< .001	5.18	[4.96, 5.40]	< .001
Within-person effects						
Perceived control$_{WP}$, γ_{10}	0.44	[0.41, 0.48]	< .001	0.45	[0.41, 0.48]	< .001
Weekend, γ_{20}	-0.09	[-0.25, 0.07]	.268	-0.09	[-0.25, 0.07]	.258
Day of ESM, γ_{30}	0.02	[-0.02, 0.06]	.325	0.02	[-0.02, 0.06]	.336
Between-person effects						
Perceived control$_{BP}$, γ_{01}	0.47	[0.39, 0.56]	< .001	0.45	[0.37, 0.54]	< .001
Pandemic consequences, γ_{02}	-0.08	[-0.26, 0.09]	.352	-0.16	-0.30, -0.02]	.023
Extraversion, γ_{03}	0.27	[-0.13, 0.68]	.184	0.24	[-.17, 0.64]	.251
Extraversion × Pandemic consequences, γ_{04}	0.23	[-0.14, 0.61]	.227	0.12	[-0.21, 0.45]	.486
Age, γ_{05}	0.12	[-0.03, 0.27]	.123	0.13	[-0.02, 0.27]	.093
Gender, γ_{04}	0.49	[-0.03, 1.01]	.066	0.48	[-0.04, 1.00]	.072
Contact with COVID-19, γ_{03}	0.08	[-0.35, 0.52]	.705	0.08	[-0.35, 0.51]	.707
Cross-level interactions						
Perceived control × Pandemic consequences, γ_{11}	0.07	[0.04, 0.11]	< .001	0.02	[-0.01, 0.05]	.125
**Random Effects**						
Variance Intercept, $\sigma^2_{u_0}$			1.42			1.39
Variance Perceived Control $\sigma^2_{u_1}$			3.56			3.58
ICC			.28			.28
AIC			12662.629			12673.426
R^2_w/ R^2_b			.270 / .390			.271/.398

Note. *N* = 242 individuals providing 2,985 observations. Gender was coded 0 for females and 1 for males. R^2_windicates modeled variance at the within-person level, R^2_b indicates modeled variance at the between-person level. Since the model with personal consequences of the pandemic did not converge with random slopes, we fitted both models with random intercepts only for comparability. The script for the analyses with random slopes can be found on OSF.

Table S3

Exploratory By-Item Analyses for Personal Consequences Items

Variables	Item 1			Item 2			Item 3			Item 4		
	Est.	*95% CI*	*p*	*Est.*	*95% CI*	*p*	*Est.*	*95% CI*	*p*	*Est.*	*95% CI*	*p*
**Fixed Effects**												
Intercept, γ_{00}	5.16	[4.94, 5.39]	< .001	5.15	[4.93, 5.37]	< .001	5.15	[4.93, 5.38]	< .001	5.16	[4.94, 5.38]	< .001
Within-person effects												
Perceived control$_{WP}$, γ_{10}	0.44	[0.38, 0.49]	< .001	0.43	[0.38, 0.49]	< .001	0.44	[0.38, 0.49]	< .001	0.44	[0.38, 0.49]	< .001
Weekend, γ_{20}	-0.03	[-0.19, 0.12]	.662	-0.04	[-0.19, 0.12]	.658	-0.04	[-0.19, 0.12]	.655	-0.03	[-0.19, 0.12]	.664
Day of ESM, γ_{30}	0.02	[-0.02, 0.06]	.266	0.02	[-0.02, 0.06]	.276	0.02	[-0.02, 0.06]	.268	0.02	[-0.02, 0.06]	.259
Between-person effects												
Perceived control$_{BP}$, γ_{01}	0.49	[0.40, 0.57]	< .001	0.47	[0.38, 0.55]	< .001	0.48	[0.40, 0.57]	< .001	0.48	[0.40, 0.56]	< .001
Pandemic consequences, γ_{02}	0.04	[-0.06, 0.14]	.401	-0.10	[-0.19, -0.00]	.040	-0.03	[-0.12, 0.07]	.577	-0.03	[-0.14, 0.08]	.575
Age, γ_{03}	0.11	[-0.04, 0.26]	.149	0.11	[-0.04, 0.26]	.148	0.12	[-0.03, 0.26]	.130	0.12	[-0.03, 0.27]	.130
Gender, γ_{04}	0.50	[-0.02, 1.03]	.060	0.49	[-0.03, 1.01]	.064	0.52	[-0.01, 1.05]	.054	0.49	[-0.04, 1.02]	.068
Contact with COVID-19, γ_{05}	0.07	[-0.36, 0.50]	.750	0.11	[-0.32, 0.54]	.613	0.09	[-0.35, 0.52]	.692	0.09	[-0.35, 0.52]	.701
Cross-level interactions												
Perceived Control × Pandemic Consequences, γ_{11}	0.01	[-0.02, 0.04]	.396	0.02	[-0.01, 0.04]	.289	0.02	[-0.01, 0.05]	.217	0.04	[0.00, 0.07]	.034
**Random Effects**												
Variance Intercept, $\sigma^2_{u_0}$			1.47			1.45			1.48			1.47
Variance Perceived Control, $\sigma^2_{u_1}$			0.06			0.06			0.06			0.06
Residual Variance, σ^2_e			3.34			3.33			3.34			3.33
ICC			.34			.33			.34			.34
AIC			12596.568			12592.692			12596.189			12593.189
R^2_w/ R^2_b			.296 /0.378			.299/.386			.295/.376			.296/.376

Note. *N* = 242 individuals providing 2,985 observations. Item 1= having fewer contacts with friends; Item 2 = having trouble to focus on schoolwork; Item 3 = avoiding social contacts out of fear of infection; Item 4 = doing well with avoiding contact with others (reversed). Gender was coded 0 for females and 1 for males. R^2_windicates modeled variance at the within-person level, R^2_b indicates modeled variance at the between-person level.

Table S4

Exploratory By-Item Analyses for Societal Consequences Items

**Variables**	**Item 1**			**Item 2**		
	Est.	*95% CI*	*p*	*Est.*	*95% CI*	*p*
**Fixed Effects**						
Intercept, γ_{00}	5.16	[4.94, 5.39]	< .001	5.19	[4.97, 5.40]	< .001
Within-person effects						
Perceived control$_{WP}$, γ_{10}	0.44	[0.38, 0.49]	< .001	0.44	[0.38, 0.49]	< .001
Weekend, γ_{20}	-0.03	[-0.19, 0.12]	.697	-0.04	[-0.19, 0.12]	.655
Day of ESM, γ_{30}	0.02	[-0.02, 0.06]	.269	0.02	[-0.02, 0.06]	.271
Between-person effects						
Perceived control$_{BP}$, γ_{01}	0.49	[0.40, 0.57]	< .001	0.44	[0.36, 0.52]	< .001
Pandemic Consequences, γ_{02}	0.04	[-0.06, 0.15]	.403	-0.24	[-0.34, -0.13]	< .001
Age, γ_{03}	0.11	[-0.04, 0.26]	.162	0.14	[-0.01, 0.28]	.064
Gender, γ_{04}	0.49	[-0.04, 1.01]	.069	0.44	[-0.06, 0.95]	.085
Contact with COVID-19, γ_{05}	0.07	[-0.37, 0.50]	.768	0.02	[-0.40, 0.44]	.925
Cross-level interactions						
Perceived Control × Pandemic Consequences, γ_{11}	0.03	[-0.00, 0.06]	.070	-0.01	[-0.05, 0.02]	.401
**Random Effects**						
Variance Intercept, $\sigma^2_{u_0}$			1.47			1.33
Variance Perceived Control, $\sigma^2_{u_1}$			0.06			0.06
Residual Variance, σ^2_e			3.34			3.33
ICC			.34			.32
AIC			12594.027			12578.610
R^2_w/ R^2_b			.295 /.377			.316/.427

Note. *N* = 242 individuals providing 2,985 observations. Item 1 = being anxious/worried, Item 2 = believing that we as a society can get the situation under control (reversed). Gender was coded 0 for females and 1 for males. R^2_w indicates modeled variance at the within-person level, R^2_b indicates modeled variance at the between-person level.
